# PERGOLA: fast and deterministic linkage mapping of polyploids

**DOI:** 10.1186/s12859-016-1416-8

**Published:** 2017-01-04

**Authors:** Fabian Grandke, Soumya Ranganathan, Nikkie van Bers, Jorn R. de Haan, Dirk Metzler

**Affiliations:** 1 Genetwister Technologies B.V., Wageningen, The Netherlands; 2 Fakultät für Biologie, University of Munich (LMU), Munich, Germany

**Keywords:** Linkage mapping, Polyploids, Heuristic

## Abstract

**Background:**

A large share of agriculturally and horticulturally important plant species are polyploid. Linkage maps are used to locate associations between genes and traits by breeders and geneticists. Linkage map creation for polyploid species is not supported by standard tools. We want to overcome this limitation and validate our results with simulation studies.

**Results:**

We developed PERGOLA, a deterministic and heuristic method that addresses this problem. We show that it creates correct linkage groups, marker orders and distances for simulated and real datasets. We compare it to existing tools and demonstrate that it overcomes limitations in ploidy and outperforms them in computational time and mapping accuracy. We represent linkage maps as dendrograms and show that this has advantages in the comparison of different maps.

**Conclusions:**

PERGOLA can be used successfully to calculate linkage maps for diploid and polyploid species and outperforms existing tools.

**Electronic supplementary material:**

The online version of this article (doi:10.1186/s12859-016-1416-8) contains supplementary material, which is available to authorized users.

## Background

Polyploidy describes the condition of having more than two chromosome sets and is common in flowering plants. A large share of agriculturally and horticulturally important plant species are polyploid. Among them are wheat and sugar cane, which are the most planted (∼219,046,706 ha, 2013) and most fecund (∼709,350 Hg/ha, 2013) crops, respectively [1]. In contrast to their importance, the research and tool-set for genetics in polyploids is underdeveloped. Many bioinformatics tools have been developed for diploids, but cannot be applied to polyploids (e.g. genotype calling).

Linkage mapping describes the process of calculating the genetic relation between markers. The general concept is used for decades and established in the fields of plant and animal breeding. During meiosis recombinations occur along the chromosomes. Investigating these provides information about the genetic distances of markers (e.g. SNPs). Comparing recombinations between multiple offspring in a mapping population (e.g. F2 or backcross) allows to calculate similarities between markers. The more similarly two markers co-segregate the higher the linkage is between them and the more likely it is that they are located closely together [2]. Groups of linked markers can be clustered into so called linkage groups, which ideally represent the individual chromosomes. Available linkage mapping tools for polyploids are limited to simplex and duplex markers [3–5]. Consequently, they cannot be applied to state-of-art datasets (i.e. genotyping microarray or genotyping by sequencing (GBS) data). A linkage map can be used to detect quantitative trait loci (QTL).

We developed PERGOLA, a linkage mapping tool for polyploids implemented as R package (https://cran.r-project.org/package=pergola) [6]. We demonstrate its application to simulated and real data sets of varying ploidy types and levels. The results for simulated data are deterministic and produce the correct linkage map. We further validate this with systematic simulations. Application to real data sets and comparison to three existing tools shows the advantages of our method. The transformation of linkage maps into dendrograms allowed us to compare the results visually and computationally.

PERGOLA is much faster than existing mapping tools and therefore also provides an alternative for linkage mapping in diploids.

## Methods

### Data

We applied PERGOLA to simulated and real datasets of varying ploidy levels. We simulated a hexaploid F2 population with 100 offspring with PedigreeSim [7]. The input linkage map was designed similar to the chromosomal characteristics of rose, a polyploid species for which a linkage map is available. This map consists of seven linkage groups with lengths of 75, 110, 85, 100, 110, 95 and 80cM [8]. We simulated the dataset with randomly distributed markers. We randomized the order of markers and alleles for each sample-marker pair to remove any prior knowledge that is not available for a real dataset. We systematically changed 0, 0.1, 0.2 and 0.3 of the genotypes to both missing and wrong genotypes (e.g. AAATTT → AAAATT). Each of the 16 configurations was repeated 100 times.

The allotetraploid data set was obtained from a peanut experiment with 89 offspring [9]. The population’s parents are doubled haploid, thus the offspring behave similar to diploids. The dataset consists of 459 markers organized in ten linkage groups. 3,101 of the 40,851 genotypes are missing. The autotetraploid data set consists of 156 samples of the MSL603 potato population [10]. For our linkage map we used a subset of markers where both parents were heterozygous (AABB).

### Linkage mapping

**Fig. 1:**
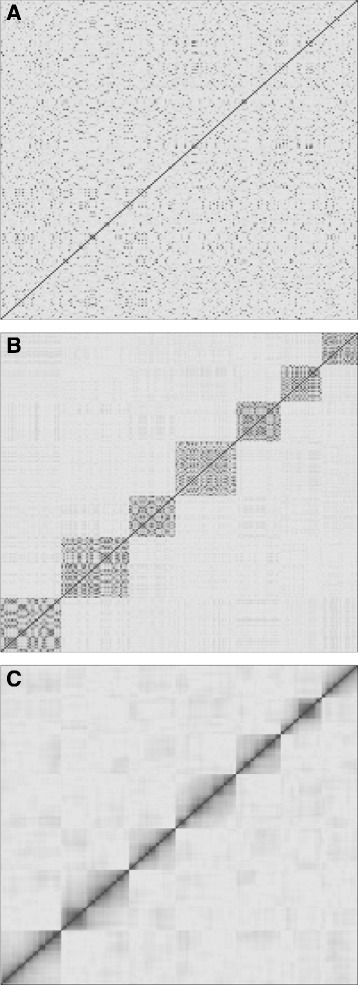
Ordering of recombination matrix. The three stages of ordering (**a**, **b**, **c**) visualized with the pairwise recombination frequency matrix. Each row and column represents one marker. Dark and light shades of grey indicate low and high recombination frequencies, respectively. **a**) The markers are in random order. The diagonal is dark because the recombination frequency of a matrix with itself is zero. **b**) The markers are ordered according to their linkage groups. Seven separated rectangles are formed and easy to distinguish. **c**) The markers are ordered within each linkage group. Most of the low values moved to the diagonal

Similarity of genotypes was used to predict recombination frequencies and linkage between markers. This information was then used to estimate linkage maps. Linkage mapping was divided into the steps grouping, ordering, and spacing. The former two are visualized in Fig. 1.

#### Recombination

Genetic recombination describes the exchange of DNA between two chromosomes during meiosis. The recombination frequency *θ* of two markers (e.g. SNPs) is the frequency of one crossover between them. The concept of linkage mapping differs for polyploids because the calculation of recombination frequencies is more complex than for diploids [11]. In the past there have been multiple approaches to this problem [12–14]. All calculate the recombination frequency exactly, but to our knowledge none of them has resulted in a tool. We, on the contrary, estimate the pairwise recombination frequency *θ* between two markers *m* and *n* of ploidy *p* with

1 $$ \hat{\theta}_{m,n} = \frac{\min(A_{m,n}, B_{m,n})} {A_{m,n} + B_{m,n}}  $$

where *A* and *B* are sums of recombination events for the two possible allelic configurations defined as $A_{m,n} = \sum _{i}{\lvert m_{i} - n_{i}\rvert }$ and $ B_{m,n} = \sum _{i}{\lvert m_{i} - p + n_{i}\rvert }$. *m*_*i*_ and *n*_*i*_ are the allele counts for individual *i* for each pair of markers *m* and *n*. For instance the allele counts at tetraploid loci AAAA, AABB and ABBB would be 4,2 and 1, respectively. The two different allelic configurations account for the unknown parental origin of the alleles. Two markers *m*=*A* *A* *A* *A* (4) and *n*=*A* *A* *A* *T* (3) indicate *A*_*m*,*n*_=1 and *B*_*m*,*n*_=3 recombination events and $\hat {\theta } = \frac {1}{4}$ in this example. Consequently, $\hat {\theta }$ never exceeds 0.5, which is a requirement for recombination frequencies in linkage mapping [15].

Our heuristic approach is fast and ignores some biological details (e.g. double reduction) [16]. PERGOLA can make use of large numbers of both markers and samples, provided in modern high throughput datasets. High marker density results in long chains of markers in linkage disequilibrium. Consequently multiple recombination events between two neighboring markers in our mapping populations become very rare and can be ignored. The large number of samples provides a high resolution of recombinations. Accordingly we can even distinguish between markers with very high linkage. Table 1 shows the minimal number of recombinations between pairs of markers.

**Table 1:** Observable numbers of recombination events between two biallelic tetraploid markers

	AAAA	AAAB	AABB	ABBB	BBBB
AAAA	0	1	2	3	4
AAAB	1	0	1	2	3
AABB	2	1	0	1	2
ABBB	3	2	1	0	1
BBBB	4	3	2	1	0

A and B are the major and minor alleles, respectively. Higher numbers of recombination are possible due to double crossovers (i.e. AAAA /AAAA could be 2 or 4), but ignored by the heuristic

The heuristic calculation of recombination frequencies overestimates linkage because it always assumes the lowest possible number of recombinations. This is not necessarily the actual number of recombination events. For instance AABB/AABB can have 0, 2, 4 or even more recombinations due to double crossovers, but we always approximate 0 in that case. If two markers are closely linked we assume no recombination and the approximation is correct. For distant markers the genotypes are different for a large proportion of the population by chance and their $\hat {\theta }$ will be larger although we assume no recombination for some individuals. The increased number of genotypes in polyploids compared to diploids provide a higher resolution of recombinations and improves the heuristic approach.

#### Grouping markers

A linkage group is a subset of co-segregating markers. Ideally each linkage group represents one chromosome, but that cannot always be achieved [17]. Markers with a recombination frequency $\hat {\theta }$ below a certain threshold are grouped. The threshold depends on the dataset and should be adjusted manually. PERGOLA groups the markers based on hierarchical clustering with single linkage distance [18]. Single linkage ensures that markers with the lowest recombination frequency end up in the same group and are not affected by markers on the other end of the chromosome, which would be the case for complete or average linkage. The approximated recombination frequencies are used as distances. The default values might not be suitable for all species or data sets. Thus, the result of the clustering should be manually inspected. Datasets with a low number of samples can result in an overestimated count of linkage groups. Some of these might contain a very low amount of markers and should be filtered out. In our implementation of PERGOLA the default filter threshold is 0.05. Hence, each linkage group should contain at least 5 percent of the markers. It needs to be decreased if the chromosome number is larger than 10 or if the markers are not distributed evenly. We recommend to visualize the data in form of a dendrogram as well as a heatmap. Dendrograms provide information about the distances between the linkage groups. Large differences in height indicate a good resolution. If branches of high height have a single leaf they should be filtered out. The heatmap visualization supports the comparison of the linkage groups’ sizes. Each linkage group is represented by a rectangle. The sharper the edges of the rectangle and the less recombination is indicated outside of them the better is the grouping (compare Fig. 1).

#### Marker ordering

PERGOLA applies the optimal leaf ordering (OLO) algorithm to determine the marker order within each linkage group [19]. First, one dendrogram is calculated for each group, based on the estimated recombination frequencies $\hat {\theta }$ by using a single-linkage hierarchical clustering. Markers are organized as leafs of the dendrogram and branches represent the relationships between them. Second, OLO optimizes the ordering of the dendrogram’s leafs without changing the hierarchical clustering by recursively calculating the optimal subtree orientation of the *n*−1 internal nodes. The decision whether a node is flipped or not is based on the best global ordering. Each tree has 2^*n*−1^ possible orderings where *n* is the number of leafs. OLO finds an order that minimizes the sum of adjacent recombination frequencies (SARF) with a worst-case complexity of *O*(*n* ^4^) [19]. Assuming that SNPs have the ordering *s*=(1,2,3,...,*n*), the SARF criterion is defined as

2 $$  SARF = \sum\limits_{i=1}^{n-1} \hat{\theta}_{a_{i}a_{i+1}}  $$

where $\hat {\theta }_{a_{i}a_{i+1}}$ is the estimated recombination frequency between a SNP *a*_*i*_ and its adjacent SNP *a*_*i*+1_ [20]. The subscripts *i* and *i*+1 identify the SNPs in order *s*. OLO includes an early termination step, which avoids unnecessary calculations, if the result cannot be improved. That usually reduces the runtime, but the worst case remains unchanged.

Given high marker density datasets the marker order according to the SARF criterion is not always unique. Multiple close markers or single distant markers can result in varying linkage maps with the same SARF value. Subsequently the same input leads to different results, which is the definition of a non-deterministic algorithm. In these cases we stepwise extend the SARF criterion to neighboring markers until the ordering is resolved unambiguously. For real-world datasets our extension leads to unique results, but theoretically it is possible to construct worse-case scenarios, where only ambiguous orders can be found. This size of the neighborhood *l* is indicated as subscripted number:

3 $$ SARF_{l} = \sum\limits_{k=1}^{l}\sum\limits_{i=1}^{n-l} \hat{\theta}_{a_{i}a_{i+k}}  $$

where $\hat {\theta }_{a_{i}a_{i+k}}$ is the estimated recombination frequency as described for Eq. 2. We identified two cases where the extension of the SARF criterion is required to obtain a deterministic solution.

- Two markers have equal distances to their neighbors, but a smaller distance between each other. These can be swapped without changing the SARF value (Markers *C* and *D* in Table 2).

**Table 2:** Pairwise distance between the six markers A-F

	A	B	C	D	E
B	2				
C	4	**4**			
D	6	**4**	2		
E	8	7	**4**	**4**	
F	12	10	7	5	3

The bold values indicate equal distances to neighboring markers and thus, ambiguous marker orders. The underlined values are taken into account in the ordering step of PERGOLA to obtain a deterministic result
- A marker can be placed at both ends of a linkage group. Its similarity is high enough to be in the cluster, but it is an outlier within the linkage group. Therefore, it is placed at one of the edges.

An example is provided in Table 2. The orders *s*_1_=(*A*,*B*,*C*,*D*,*E*,*F*), *s*_2_=(*A*,*B*,*D*,*C*,*E*,*F*) and *s*_3_=(*B*,*A*,*C*,*D*,*E*,*F*) have the same *S* *A* *R* *F*_1_ value of 15. Extending the neighborhood *n* to 2 leads to *S* *A* *R* *F*_2_(*s*_1_)=32, *S* *A* *R* *F*_2_(*s*_2_)=36 and *S* *A* *R* *F*_2_(*s*_3_)=34. Thus, *s*_1_ is the best order and *s*_2_ and *s*_3_ can be discarded.

The determinism nature of our method refers to the outcome of the marker ordering step. The general process of linkage mapping is still a stochastic approximation of the real linkage based on an SNP markers.

#### Marker spacing

For spacing we applied the Haldane mapping function [21] to the recombination frequencies. Our implementation includes the Kosambi and Carter-Falconer mapping functions as alternatives.

### Comparison

We compare PERGOLA with three other linkage mapping tools:

- JoinMap®; 4.1 by Kyazma®; B.V., Wageningen, Netherlands [22] is one of the most popular linkage mapping tools [18].
- MapMaker Macintosh version 2.0 [23] was used by the authors of the peanut dataset [9].
- R/qtl version 1.33-7 is an R package that supports linkage mapping [24].

Further we recalculate the maps of hexaploid simulated data and autotetraploid potato data. In addition to visual comparison we applied two computational correlation measurements. Our first method to compare two dendrograms is the Goodman-Kruskal-gamma index [25]. It calculates the tree similarity by rank comparison of all *n* ^2^ pairs of markers. The second method is cophenetic correlation [26]. It measures the similarity of pairwise distances between all markers. Both correlation measures lie between -1 and 1, indicating negative and positive correlation, respectively.

## Results and discussion

### Linkage maps of simulated data

**Fig. 2:**
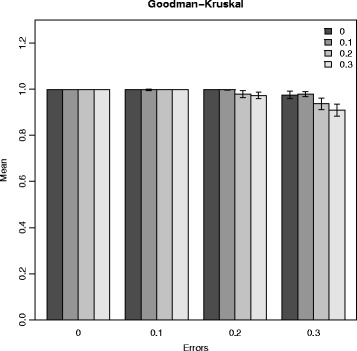
Goodman-Kruskal correlations. Goodman-Kruskal correlation values of simulated hexaploid data sets and corresponding linkage maps generated by PERGOLA. The x-axis shows four groups with different error values, indicating the amount of errors introduced to the data. The y-axis shows the mean Goodman-Kruskal correlation value for 100 simulations per parameter combination. The standard errors are represented by bars. Each group consists of four differently colored bars, indicating different rates of missing values

We applied PERGOLA to simulated hexaploid datasets. First, the datasets were randomized to remove any prior information (e.g. haplotypes) that is not available for a real dataset. Second, we calculated the pairwise recombination frequencies for all markers. An example is visualized in Fig. 1. The randomized order of the markers is replaced by a hierarchical clustering order based on the recombination frequencies. The rectangles in the heatmap have sharp edges and suggest seven linkage groups. This is consistent with the seven chromosomes in the linkage map that we used to simulated the data. In the next step, PERGOLA orders the markers within the seven linkage group unambiguously. We calculate the spacing between markers based on the recombination frequencies and thus obtain our final linkage map. This map and the initial input map of PedigreeSim are transformed into dendrograms to make them comparable. Next, we calculate the Goodman-Kruskal index and cophenetic correlation values. This is repeated 100 times for each simulation parameter combination. The Goodman-Kruskal correlation values are shown in Fig. 2. For error free input data the linkage maps are equal independent of missing values. The higher the error rate, the more impact has the proportion of missing values. In general, the values are close to one indicating good linkage maps. The cophenetic correlation results are shown in the Additional file 1. Again the values strongly correlate with the proportions of missing and erroneous data. The values are not as high as the Goodman-Kruskal correlations because the cophenetic correlation is more sensitive.

The results should be interpreted with caution because the data is simulated. PedigreeSim simulates the genotypes based on one model, which has been intensively discussed in the community [7, 27, 28]. Alternative simulation models (e.g. Rehmsmeier, 2013 [12]) might lead to differing results. The models differ in two main aspects: First, PedigreeSim uses a descriptive model, which explains the probability distribution of gamete modes as observables (e.g. recombination / no recombination). In contrast, other models are analytical and explain the distribution with meiotic mechanisms. Second, PedigreeSim calculates recombination rates and double reduction independently, while alternative models treat them as reliant [12]. The differences are limited to the simulation of autopolyploids because they only occur during quadrivalent meiosis. Further, they exchange single alleles after crossing-over events in case of double reduction, but the other alleles remain the same.

### Application to real allotetraploid data

**Fig. 3:**
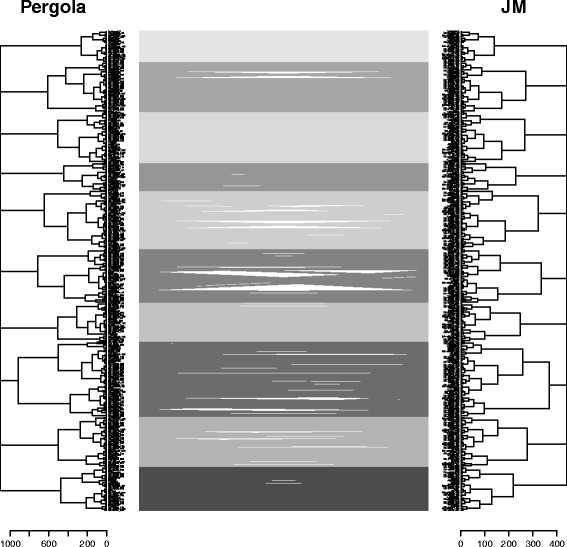
Global linkage map comparison - PERGOLA and JoinMap®;. Comparison of the linkage map created by PERGOLA and JoinMap®;. Both are split into ten linkage groups, highlighted by different shades of gray. The linkage groups consist of the same markers. White spaces indicate differences in the marker ordering

We applied PERGOLA to allotetraploid genotypes of a peanut crossing population [9]. The dataset originates from doubled haploid (DH) pedigrees and behaves similar to diploids. Application of PERGOLA resulted in a linkage map consisting of ten linkage groups (see left side of Fig. 3). That matches the expected number of chromosomes for peanut known from literature [29]. Further validations are difficult because the real linkage map is unknown.

However, the diploid nature of the peanut dataset allowed us to compare the results and performance of PERGOLA to linkage mapping tools, which do not support polyploids. We selected MapMaker, JoinMap®; and R/qtl. MapMaker was used by the authors of the peanut dataset [9] and the results are publicly available. Runtimes are not provided by the authors and would not be informative as the computational setup is not comparable. JoinMap®; is one of the most popular linkage mapping tools [18]. However, it is neither open-source, nor open-access and only works on Windows systems. R/qtl is publicly available as R-package and allows reproduction of our comparison. More linkage mapping tools are available, but software comparison is not the main subject of this publication.

Comparing linkage mapping tools is difficult because depending on the parameter settings each tool can output different maps. We used the default parameters of each tool and the Haldane function to calculate the spacing between the markers. The results gave a general impression of the performance and should not be overinterpreted. All tools could be applied in multiple ways and lead to different maps. The motivation of the comparison was to find out if PERGOLA performs worse than the other tools for a diploid-like data set. For polyploid data sets the other tools can not be applied and PERGOLA is the method of choice.

The runtime of MapMaker is unknown because the authors of the peanut dataset did not provide computation times. Data preparation is unique for every tool and depends on the format of the given data. Thus, we excluded that step from the time measurement. Linkage grouping was at most a matter of seconds for all methods and has been ignored. The computational time comparison focuses on marker ordering because it is the most expensive and distinctive step. In R/qtl, JoinMap®; and PERGOLA these are the commands *orderMarkers()*, *Calculate map* and *sortLeafs()*, respectively. R/qtl is the slowest one and took 16 min and 47 s. JoinMap®; had a similar runtime of 14 min and 47 s. PERGOLA was the fastest method and took 0.011 s. The better performance results from the use of the OLO algorithm compared to the sliding window approach in R/qtl and the large overhead in JoinMap®;. Runtime performance is important because linkage maps have many parameters (e.g. filter criteria) that influence the result. Faster methods allow for systematic optimization of linkage maps. For instance, usually the number of chromosomes is known. If a parameter setting results in a number of linkage maps that differs from the expected chromosome number, the setting should be changed. The runtime of PERGOLA allows for computationally expensive resampling methods (e.g. jackknifing or bootstrapping) to be used. That can improve the interpretability of linkage maps and related QTL detections.

In PERGOLA and JoinMap®; we manually selected ten linkage groups because they were suggested in the grouping step. R/qtl created these linkage groups automatically. We used the Haldane mapping function in all tools. R/qtl applies a sliding window approach where all possible permutations of markers are calculated and compared. That approach leads to locally optimized solutions, but can fail to find the best marker order within the linkage group. The default window size is seven, but performs better if the window size is increased. However, this would lead to even slower computation times and was not tested. JoinMap®; performs similar to R/qtl, but uses a more sophisticated approach. It calculates and compares different solutions internally and outputs the best solution to the user.

To compare the general linkage maps we transformed all maps into dendrograms. We aligned the chromosome orders and orientation between the maps. Dendrograms maintain the grouping, ordering and spacing of the maps and allow manual (visually) and computational comparisons. The root line connects the multiple linkage groups at the same height. In our implementation of PERGOLA its height is 0.2 times higher than the highest connection within the linkage groups. It does not reflect their similarity, but supports the readability of the map. The marker order and spacing in the map equal the leaf order and branch height in the dendrogram. We created tanglegrams from the dendrograms for a pairwise comparison of all maps [30]. They allow us to observe differences in the grouping, i.e. whether the same set of markers is in the same linkage groups. The branching height in the dendrogram provides information about the spacing. Traditionally linkage maps are represented as bars or lines. Each bar represents one linkage group from one map. Lines between the bars indicate the rearrangements between two maps. The linkage groups are distributed so that collisions are minimized. However, for large numbers of linkage groups and high marker density maps, that representation is difficult to interpret. The transformation into a tanglegram is possible without a loss of information, but with a gain in clarity. The spacing information moves into the horizontal dimension and can be explored separately. Markers which are not included in both maps are not shown because they do not contribute to comparison. However, their number should be provided along with the tanglegram. An example tanglegram is shown in Fig. 3 and others are provided in the Additional file 1.

The pairwise tanglegrams show that the maps are generally similar. All maps consist of ten linkage groups, mainly containing the same markers. The maps by R/qtl and MapMaker contain five and six markers more than PERGOLA and JoinMap®;, respectively. This information is not illustrated in the tanglegrams. The markers have been filtered out and could not be integrated into the ten linkage groups. The total number of markers in the dataset is 459. It is unknown how many have been filtered out for the MapMaker map because they have not be provided together with the map. However, the marker density is not significantly reduced by the filtering. The quality of the map is more important, than a small number of additional markers. Thus, noisy markers should be filtered out rather than creating large gaps in a linkage group.

In our experiment, the Goodman-Kruskal-gamma index for all pairs of maps is almost 1, indicating perfect correlation. This contradicts the observations we made in the tanglegrams where we observe differences between the linkage maps. Marker grouping has a much larger effect on the Goodman-Kruskal-gamma index than ordering or spacing and if many markers are grouped similarly, differences in the latter steps are not represented by it. We conclude that the Goodman-Kruskal-gamma index is too insensitive for the allotetraploid data set. This is also supported by our simulation study. In contrast the cophenetic correlation coefficient provides reasonable measurements between the maps, as shown in Table 3.

**Table 3:** Pairwise correlations between the four maps. The bottom and top triangles show cophenetic and Goodman-Kruskal correlations, respectively

	PERGOLA	R/qtl	JoinMap®;	MapMaker
PERGOLA	-	0.999	0.999	0.999
R/qtl	0.930	-	0.999	0.999
JoinMap®;	0.938	0.922	-	0.999
MapMaker	0.961	0.928	0.915	-

The results show that PERGOLA calculates linkage maps in a fraction of the time of the other methods. That makes it not just a useful method for polyploid crops, but also as an alternative for diploid datasets. The heuristic approach of the recombination calculation leads to minor rearrangements in the grouping. They can be neglected given the overall map similarity and performance advantages of PERGOLA. The tanglegrams suggest a higher similarity between R/qtl, JoinMap®; and MapMaker because the grouping is identical. On the contrary, the cophenetic correlation indicates that the map by JoinMap®; is more similar to the PERGOLA map. That supports our aforementioned hypothesis, that there is not one correct linkage map and we can only estimate the biological situation from different directions. Depending on the input data, filtering parameters, linkage mapping tools and validation criteria, multiple maps are valid. Currently, it is impossible to discard one map or choose one over the other.

**Fig. 4:**
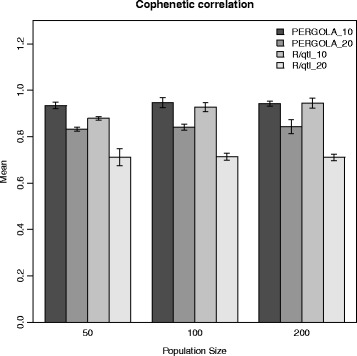
Diploid simulation study result. We simulated six setups of diploid populations with two chromosomes and repeated each 100 times. We used population sizes of 50, 100 or 200 and 10 or 20 markers per chromosome. We applied PERGOLA and R/qtl to calculate linkage maps which were compared with the reference map. The bars show the mean correlation value of 100 repetitions and the error bars indicate the standard error

We conducted a simulation study to validate the results of PERGOLA and R/qtl for diploids where the real map is known. JoinMap®; was excluded because it is limited to a graphical interface and could not be automatically applied to the hundreds of simulated datasets. We used two different numbers of markers (10 and 20 per chromosome) and three population sizes (50, 100, 200), which resulted in 6 different combinations per tool. Each combination was repeated 100 times. The input linkage maps consisted of two chromosomes and randomly spaced markers. We compared the reference maps with the calculated ones using cophenetic and Goodman-Kruskal correlation. The mean values and standard errors of the cophenetic correlation are shown in Fig. 4. PERGOLA and R/qtl perform similarly for 10 marker maps independently of the population size. For setups with 20 markers per chromosome the sliding window approach of R/qtl reaches its limits and the linkage maps differ significantly. Taken together, PERGOLA performs better not only computationally, but also produces better linkage maps for diploids.

### Application to real autotetraploid data

We did another map comparison with the second real data set, a population of 190 offspring from an autotetraploid potato cross [31]. The authors created a linkage map by combining JoinMap®; and customized scripts for the calculation of recombination frequencies. Their procedure includes multiple runs of JoinMap®; therefore the performance is even worse than by using JoinMap®; itself. Comparison of our map to the published one results in 0.962 and 0.949 for the cophenetic correlation and Goodman-Kruskal index, respectively. We performed a permutation test to validate the statistical significance of the Goodman-Kruskal-gamma index for the given dendrogram. Our null-hypothesis was that the two dendrograms are stochastically independent. After 100 random permutations all values were close to zero and lower than our value of 0.949 (Additional file 1). We rejected the null-hypothesis and concluded that the two dendrograms are dependent and show some similarity. The Goodman-Kruskal-gamma index was more sensitive for the autotetraploid dataset because it consists of less markers than the allotetraploid dataset and differences in the marker ordering were not covered by similar marker grouping. Similarly, we performed a Mantel permutation test to assess the significance of the cophenetic correlation. 99 permutations resulted a Monte-Carlo *p*-value of 0.01 and similar results as the previous permutation test (Additional file 1). Again, we reject the null-hypothesis and conclude dependence of the dendrograms.

## Conclusions

PERGOLA allows the creation of linkage maps for polyploid crops. The application to simulated data showed that it leads to reasonable linkage maps. Further, we demonstrated that it can be successfully applied to real datasets with different polyploidy types. PERGOLA outperformed existing programs for diploids in terms of computation time and mapping accuracy. The transformation of linkage groups into a two dimensional dendrogram has been shown to be a valuable alternative to the currently dominating bar scheme. It is more structured and allows to evaluate the three steps of grouping, ordering and spacing separately. The Goodman-Kruskal index is too insensitive to compare linkage maps and the cophenetic correlation index should be used instead. Taken together, PERGOLA is a valuable extension not only to the polyploid genetic toolbox, but for geneticists in general.

## Additional file

Additional file 1 Supplementary image. PDF including the additional tanglegrams and results of the simulation study. (PDF 355 kb)

## Abbreviations

DH: Doubled haploid
GBS: Genotyping by sequencing
OLO: Optimal leaf ordering
QTL: Quantitative trait loci
SARF: Sum of adjacent recombination frequencies
