# Supplementary material for: PERGOLA: fast and deterministic linkage mapping of polyploids
Source: BMC Bioinformatics. 2017 Jan 4;18:12. doi: 10.1186/s12859-016-1416-8 (PMC5210299; doi:10.1186/s12859-016-1416-8)
Supplement: Additional file 1 — Supplementary image. PDF including the additional tanglegrams and results of the simulation study. (PDF 355 kb) [file 12859_2016_1416_MOESM1_ESM.pdf]

Supplementary materials

Grandke et al.

August 28, 2016

Contents

1 Introduction **1**

2 Allotetraploid data **2**

3 Simulated data **7**

4 Autotetraploid data **9**

1 Introduction

This document provides the supplementary materials for **Pergola: Fast and Deterministic Linkage Mapping of Polyploids**.

2 Allotetraploid data

The remaining 5 tanglegrams from the pairwise comparison.

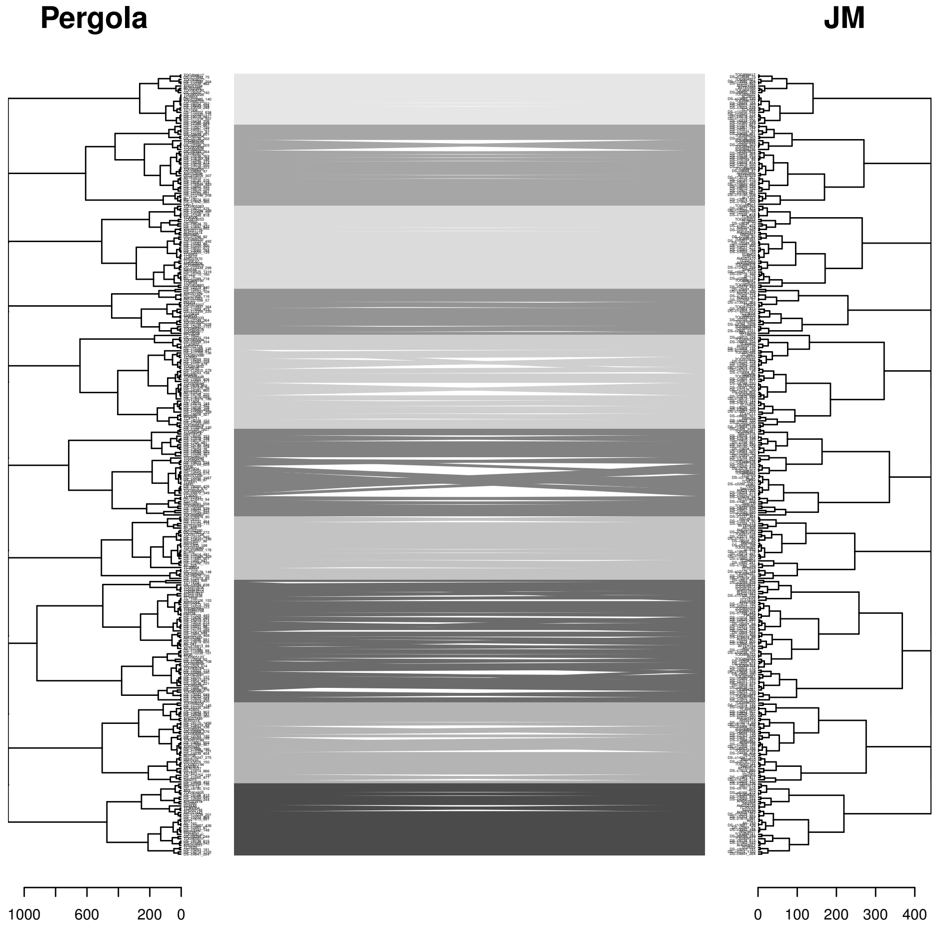

Figure 1: Comparison of the linkage map created by PERGOLA and JoinMap®. Both are split into ten linkage groups mainly consisting of the same markers. There are five rearrangements in the ordering and five linkage groups are switched.

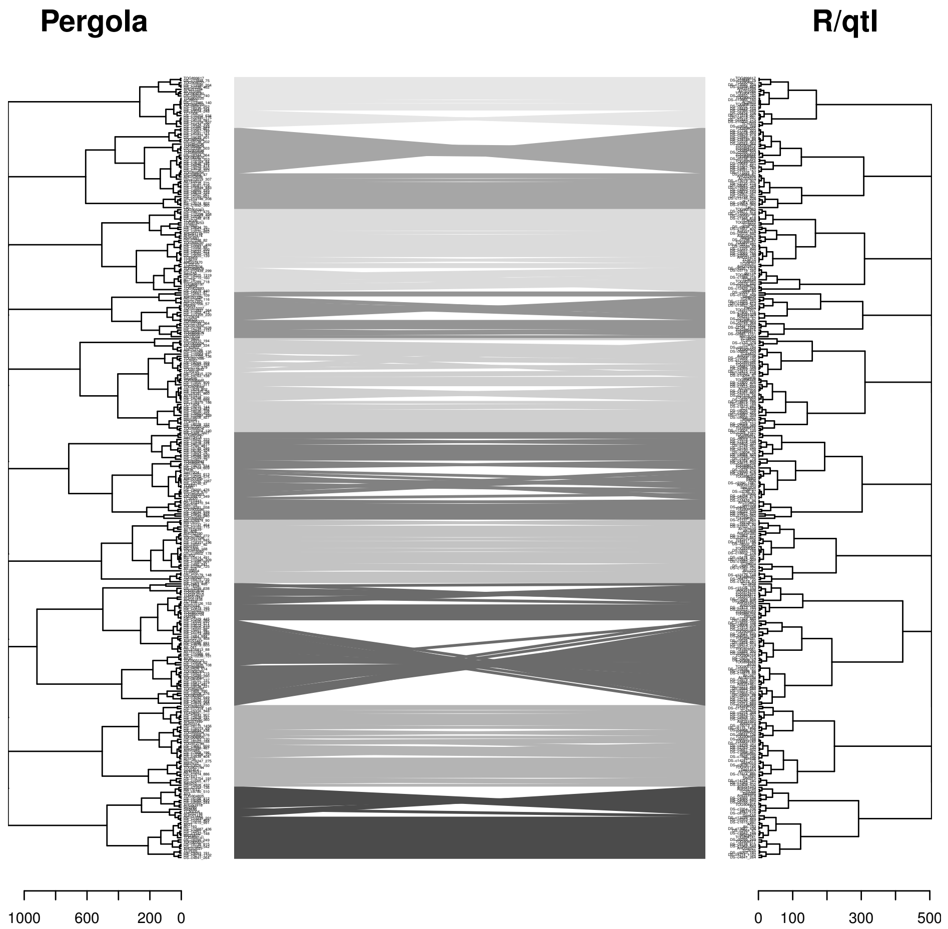

Figure 2: Comparison of the linkage map created by PERGOLA and R/qtl. Both are split into ten linkage groups mainly consisting of the same markers. There are five rearrangements in the ordering and five linkage groups are switched.

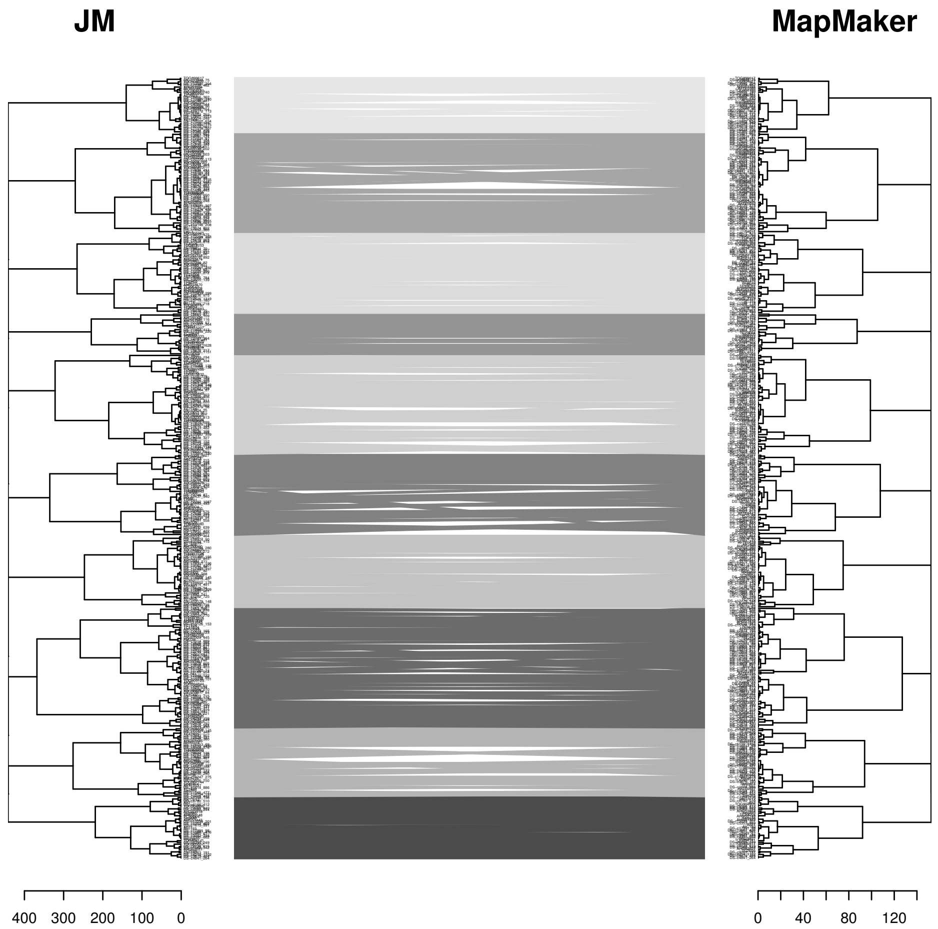

Figure 3: Comparison of the linkage map created by JoinMap® and MapMaker. Both are split into ten linkage groups mainly consisting of the same markers. There are five rearrangements in the ordering and five linkage groups are switched.

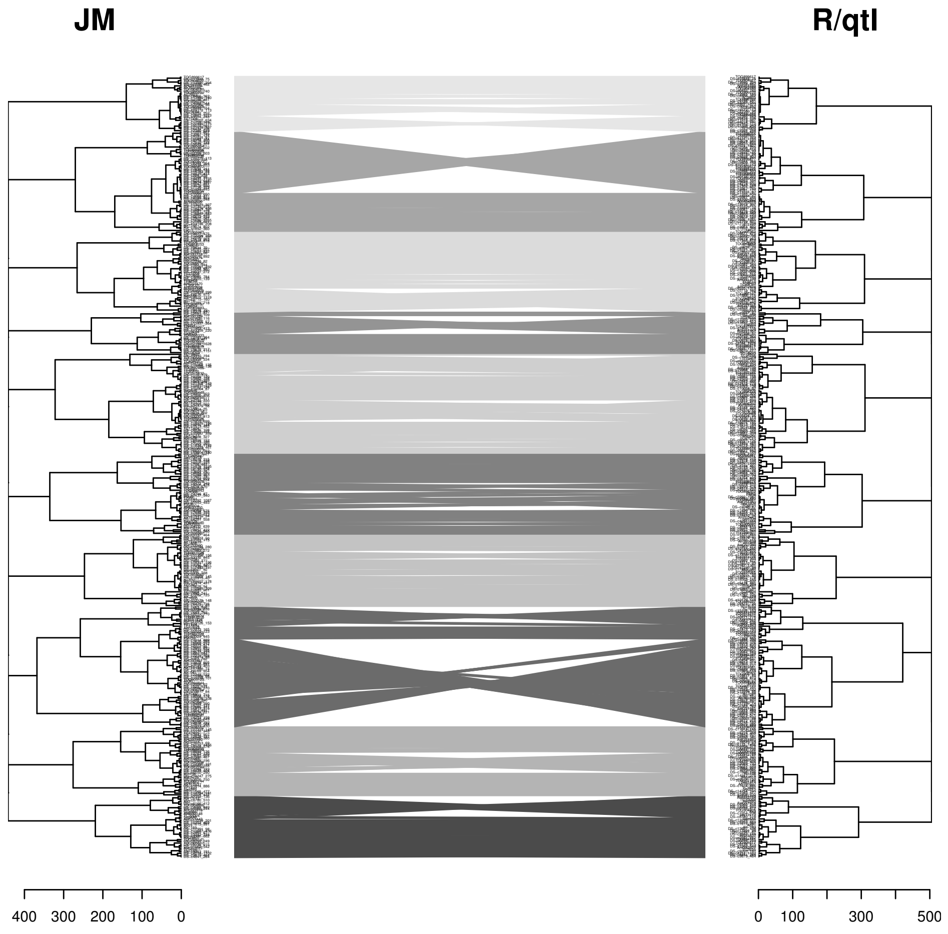

Figure 4: Comparison of the linkage map created by JoinMap® and R/qtl. Both are split into ten linkage groups mainly consisting of the same markers. There are five rearrangements in the ordering and five linkage groups are switched.

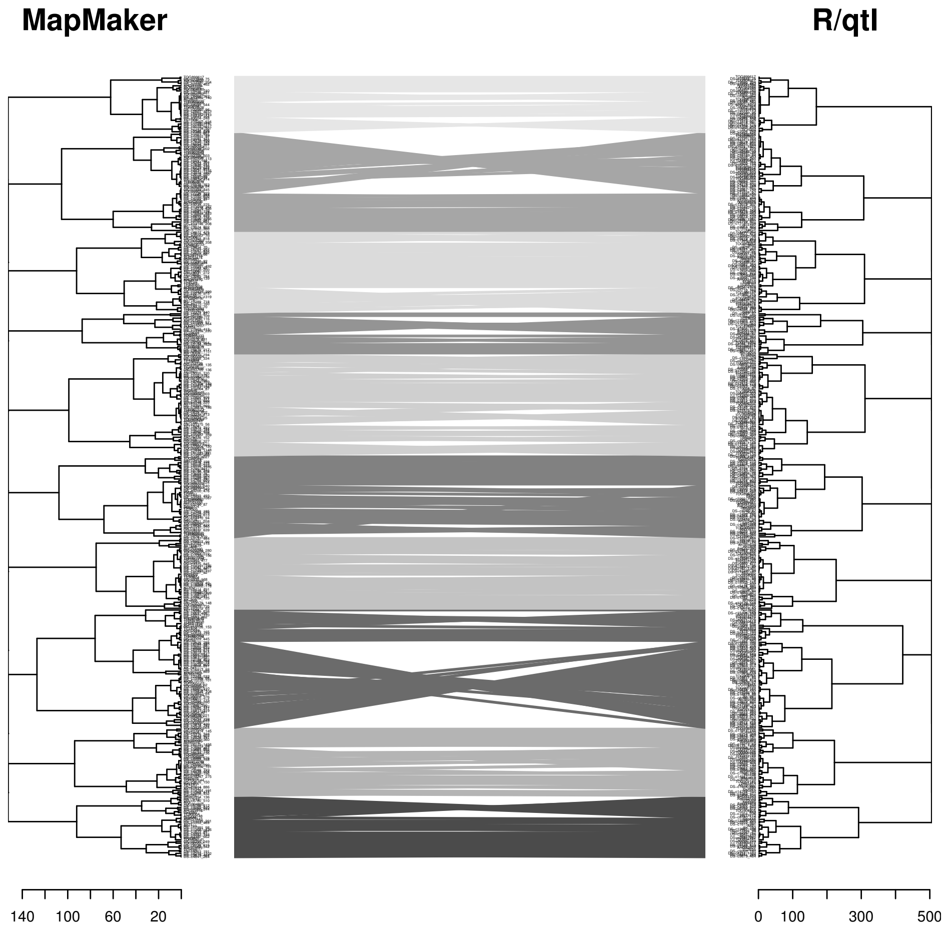

Figure 5: Comparison of the linkage map created by MapMaker and R/qtl. Both are split into ten linkage groups mainly consisting of the same markers. There are five rearrangements in the ordering and five linkage groups are switched.

3 Simulated data

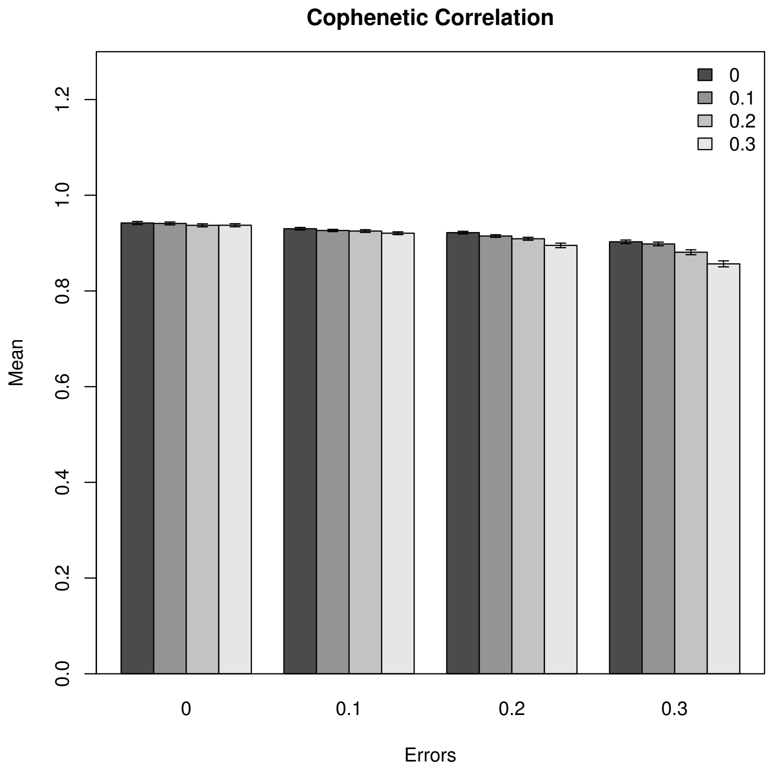

Figure 6: Cophenetic correlation values of simulated hexaploid data sets and corresponding linkage maps generated by PERGOLA as described in the main document. The x-axis shows four groups with different error values, indicating the amount of errors introduced to the data. The y-axis shows the mean cophenetic correlation value for 100 simulations per parameter combination. The standard errors are represented by bars. Each group consist of four differently colored bars, indicating different rates of missing values.)

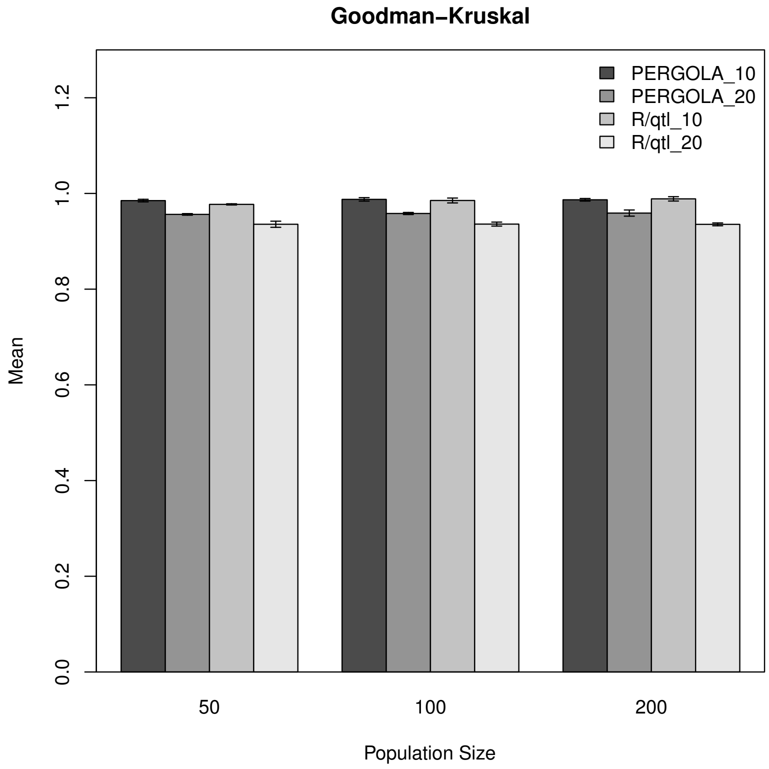

Figure 7: We simulated six setups of diploid populations with two chromosomes and repeated each 100 times. We used population sizes of 50, 100 or 200 and 10 or 20 markers per chromosome. We applied PERGOLA and R/qtl to calculate linkage maps which were compared with the reference map. The bars show the mean Goodman-Kruskal correlation value of 100 repetitions and the error bars indicate the standard error.

4 Autotetraploid data

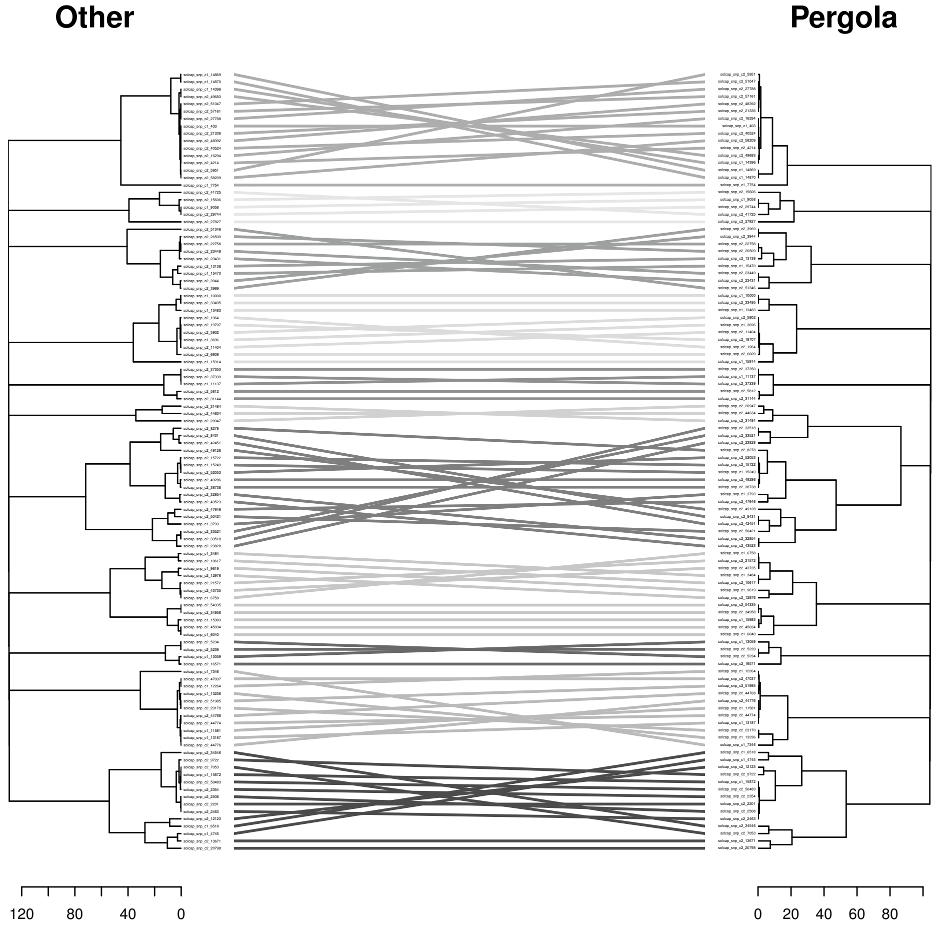

Figure 8: Comparison of the linkage map produced with a customized version of JoinMap® and PERGOLA. One PERGOLA linkage group is split into two in the other map. Otherwise the linkage groups are equal. The order is similar, but not identical.

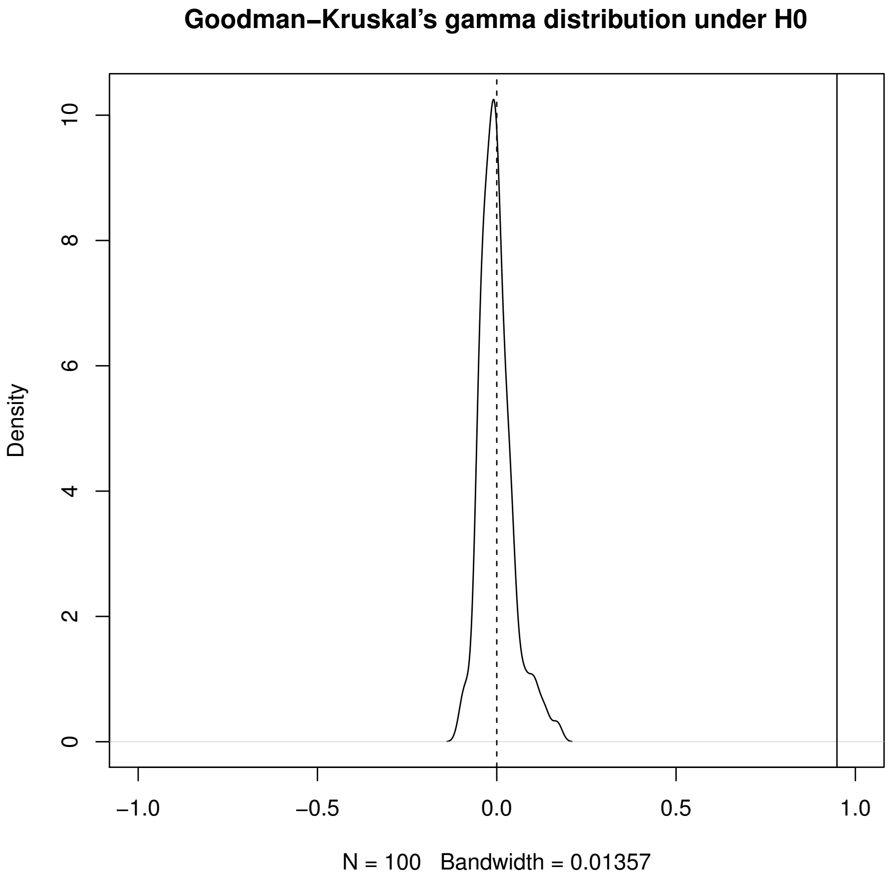

Figure 9: Result of permutation test for the dendrogram created by PERGOLA. The dendrogram is compared to itself 100 times after randomly permuting it's labels each time. The permutation test resulted in values around zero. The vertical line on the right indicates the Goodman-Kruskal index from the unpermutated data.

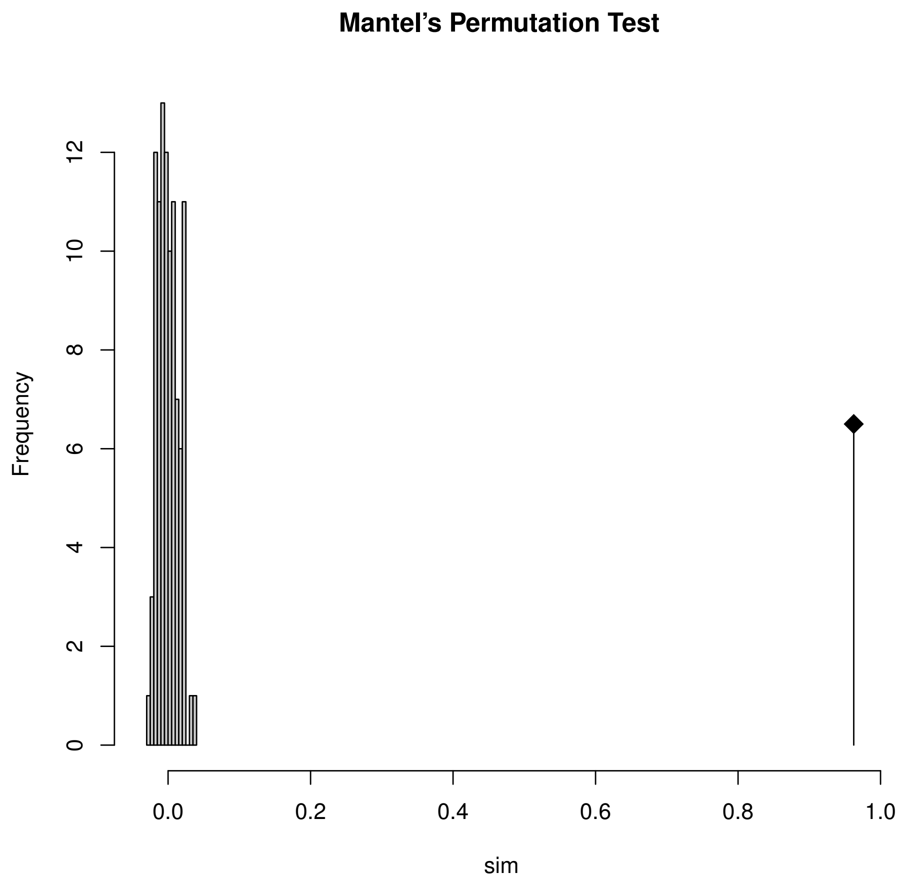

Figure 10: Result of Mantel permutation test for the dendrogram created by PERGOLA using *ade4::mantel.randtest()*. The dendrogram's cophenetic matrix is compared to the published map's cophenetic matrix 100 times after random permutation. The permutation test resulted in values around zero. The vertical line on the right indicates the cophenetic correlation value from the unpermutated data.
